# The hallmarks of hematopoietic stem cell transplantation for pediatric acute myeloid leukemia

**DOI:** 10.1038/s41375-025-02685-5

**Published:** 2025-07-09

**Authors:** Eva Rettinger, Dirk Heckl, Brenda Gibson, Martin Sauer, Dominik Turkiewicz, Katharina Kleinschmidt, Krzysztof Kalwak, Dirk Reinhardt, Franco Locatelli, Jan-Henning Klusmann, the Pediatric Diseases Working Party of the European Society for Blood and Marrow Transplantation, Eva Rettinger, Brenda Gibson, Martin Sauer, Dominik Turkiewicz, Katharina Kleinschmidt, Krzysztof Kalwak, Franco Locatelli, Jan-Henning Klusmann

**Affiliations:** 1 Department of Pediatrics, Goethe University Frankfurt, Frankfurt, Germany; 2 Institute for Experimental Pediatric Hematology and Oncology (EPHO), Frankfurt, Germany; 3 Department of Haematology and Oncology, Royal Hospital for Children, Glasgow, Scotland UK; 4 Department of Pediatric Hematology and Oncology, Hannover Medical School, Hannover, Germany; 5 Department of Pediatric Hematology, Oncology, and Immunology, Skåne University Hospital, Lund, Sweden; 6 Department of Pediatric Hematology, Oncology and Stem Cell Transplantation, University Hospital Regensburg, Regensburg, Germany; 7 Department of Pediatric Hematology, Oncology and BMT, Wroclaw Medical University, Wroclaw, Poland; 8 GPOH gGmbH—AML-BFM Trial Center, Essen, Germany; 9 Department of Pediatric Hematology and Oncology and of Cell and Gene Therapy, IRCCS Ospedale Pediatrico Bambino Gesù, Rome, Italy; 10 Catholic University of the Sacred Heart, Rome, Italy

**Keywords:** Acute myeloid leukaemia, Acute myeloid leukaemia, Risk factors

## Abstract

Allogeneic hematopoietic stem cell transplantation (HSCT) has significantly improved the outcome of children with high-risk (HR) acute myeloid leukemia (AML). Implementing allogeneic HSCT depends on numerous factors, including adverse cytogenetics, molecular abnormalities, poor response to first-line treatment, or relapsed or primary refractory disease. In HR AML, allogeneic HSCT is considered to be the consolidation strategy of choice in first complete remission (CR1) and offers the best chance of cure for patients with relapsed disease. Advances in donor/recipient typing, conditioning regimens, graft-versus-host-disease (GvHD) management, and supportive care have contributed to this improvement in overall—and transplant—outcome. This review will comprehensively discuss indications for HSCT and its modalities in pediatric AML by examining past, current, and future strategies for disease- and response-related stratification. We will examine the key importance of low/negative measurable residual disease (MRD) before transplantation and discuss conditioning regimens and graft variables, as well as novel approaches to harness the graft-versus-leukemia (GvL) effect, including targeted immunotherapy. The review will also address toxicities associated with HSCT, GvHD prophylaxis, and the management of treatment failure. Ultimately, this review seeks to inform clinical practice and highlights how improved outcomes have been achieved through the collective efforts of international study groups.

## HSCT in pediatric AML: Where do we come from?

Historically, allogeneic hematopoietic stem cell transplantation (HSCT) has been employed as a consolidation strategy in pediatric acute myeloid leukemia (AML) in first complete remission (CR1) when a human leukocyte antigen (HLA)-identical sibling donor was available [1–3]. Although a reduction in the relapse rate (RR) was observed, this benefit was offset by the risk of transplant-related mortality (TRM) [4]. Over time, optimizing disease risk stratification, pre-transplant induction or reinduction chemotherapy strategies, and supportive care resulted in significantly improved survival rates [5–7]. Studies have shown that HSCT improves overall survival (OS) and disease-free survival (DFS) compared to chemotherapy alone, largely due to a lower RR, in patients with high-risk (HR) features. As a result, both survival outcomes and disease control—at least in certain patient subsets—can be improved by HSCT [8]. This has resulted in a shift in approach, where the decision to proceed with HSCT in CR1 is now primarily driven by a risk assessment that considers disease characteristics and treatment response [4, 6, 9–12].

Despite these improvements achieved through the joint efforts of international study groups (AIEOP, BFM-AML SG, COG, JPLSG, MRC/NCRI, EORTC-CLG, NOPHO, PPLLSG, and SJCRH), the role of HSCT as a consolidation strategy for children and adolescents with newly diagnosed AML in CR1, and in particular the question as to which cytogenetics/molecular aberration constitutes HR disease [13, 14], remain controversial due to a paucity of data and the absence of randomized clinical trials comparing HSCT with other post-remission therapies [15–17].

**Fig. 1:**
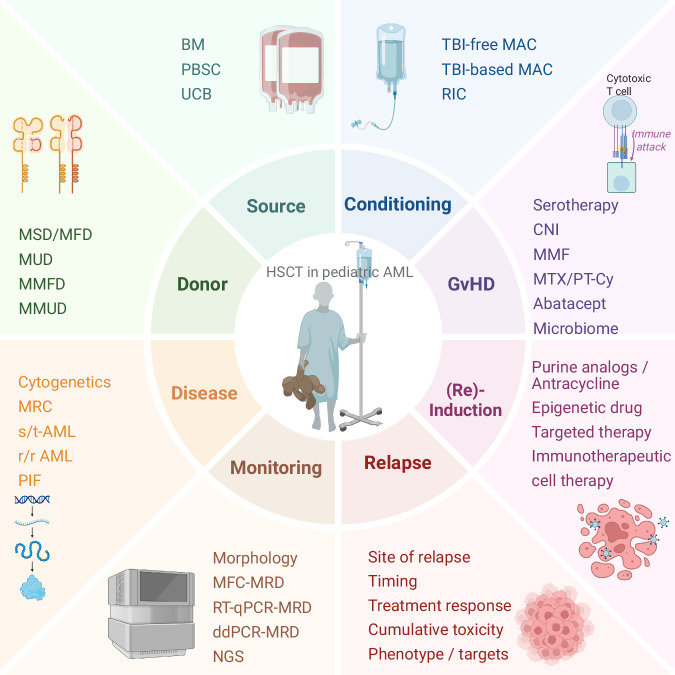
Factors affecting eligibility to and outcome of allogeneic HSCT in children with acute myeloid leukemia. Schematic representation of the variable influencing eligibility criteria, donor and graft selection, conditioning regimens, and graft-versus-host-disease prophylaxis choices in children with AML.

In this article, we present current recommendations for patient eligibility for HSCT in CR1, donor selection, stem cell graft choices, conditioning regimens, and the management of both acute and late toxicities (Fig. 1). We also discuss the importance of achieving low-level or negative measurable residual disease (MRD) prior to HSCT, as well as maintenance strategies following HSCT. Additionally, we explore potential future directions for HSCT in pediatric AML and identify key areas for further clinical research.

## To transplant or not to transplant in CR1?

The criteria for considering HSCT instead of multimodal chemotherapy alone as consolidation treatment, and for identifying which patients will benefit more from HSCT, are still debated questions. Historically, the decision was based on a disease-specific risk assessment and individual patient characteristics, and guided by a general benchmark for improved DFS. For adults, HSCT was typically considered if the expected DFS was predicted to improve by at least 10% compared to chemotherapy alone. For children, a more stringent, though not evidence-based, threshold of a 30% improvement in DFS was often used [16, 18]. Currently, the decision is generally guided by molecular and cytogenetic risk factors and treatment response. This section focuses on the current criteria indicating the need for HSCT in CR1.

## The geneticists’ point of view

**Fig. 2:**
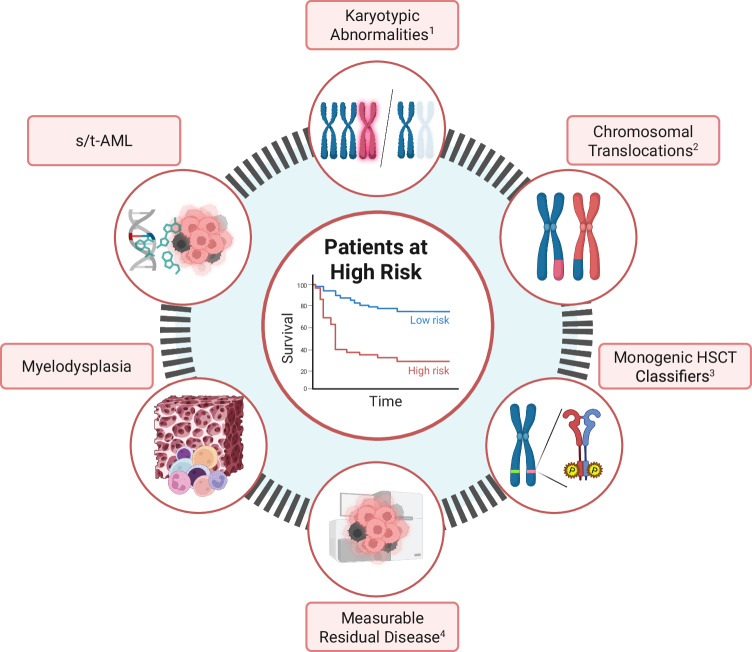
Factors indicating eligibility for HSCT in newly diagnosed pediatric AML. High-risk features in pediatric AML are defined by cytogenetic and molecular abnormalities, as well as poor response to treatment as indicated by measurable residual disease: ^1^*Karyotypic Abnormalities*: Complex karyotype (≥3 aberrations including at least one structural abnormality), excluding cases with recurrent translocations; monosomal karyotype, such as monosomy 7 or deletion 5q (-7, -5/del(5q)). ^2^*Chromosomal Translocations*: *t(16;21)(p11;q22)*→FUS::ERG; *t(9;22)(q34;q11.2)* → BCR::ABL1; *t(6;9)(p22;q34)* → DEK::NUP214; *t(7;12)(q36;p13)* → MNX1::ETV6; *inv(3)(q21q26)/t(3;3)(q21;q26)* → RPN1::MECOM; *inv(16)(p13q24)* → CBFA2T3::GLIS2; *t(5;11)(q35;p15.5)* → NUP98::NSD1; *t(11;12)(p15;p13)* → NUP98::KDM5A; 12p abnormalities; 11q23/KMT2A rearrangements, including: *t(4;11)(q21;q23)* → KMT2A::AFF1; *t(6;11)(q27;q23)* → KMT2A::AFDN; *t(10;11)(p12;q23)*→KMT2A::MLLT10. ^3^*Monogenic HSCT Classifiers*: FLT3-ITD with an allelic ratio (AR) ≥ 0.5, either alone or in combination with other recurrent abnormalities or NPM1 mutations. ^4^*Measurable Residual Disease (MRD)*: Multiparametric flow cytometry (MFD)-MRD ≥0.1% after first or second induction or (if MFC-MRD result is not available/ informative) blast count ≥5% at second induction. HR high-risk, KMT2A Histone-Lysin-N-Methyltransferase 2A, AFD Afadin, Adherents Junction Formation Factor, MLLT10 Histone Lysine Methyltransferase DOT1L Cofactor, FUS RNA Binding Protein, ERG ETS transcription factor, BCR Breakpoint cluster region, ABL1 Abelson Murine Leukemia Viral Oncogene Homolog 1, DEK protoonko-gene, NUP nucleoporin, MNX1 Motor Neuron And Pancreas Homeobox 1, ETV6 ETS Variant Transcription Factor 6, RPN1 Ribophorin I, MECOM MDS1 And EVI1 Complex Locus, FLT3 FMS-like tyrosine kinase 3, ITD internal tandem duplication, NPM1 nucleophosmin 1, WT1 Wilms Tumor 1, CBFAT3 CBFA2/RUNX1 Partner Transcriptional Co-Repressor 3, GLIS2 GLIS Family Zinc Finger 2, NSD1 Nuclear Receptor Binding SET Domain Protein 1, KDM5A Lysine Demethylase 5A, MFC multiparametric flow cytometry, MRD measurable residual disease, MRD.

Approximately 30–35% of children with AML have a cytogenetic profile that could be considered as HR and an indication for HSCT in CR1. While cytogenetics/molecular aberrations that constitute HR disease vary between cooperative study groups, there is growing consensus, and as an example, we will detail the HR cytogenetic criteria of the (Berlin—Frankfurt—Münster) AML-BFM study group in this section (Fig. 2) [15, 19–22]. Genetic abnormalities considered HR have evolved over time and will continue to evolve, and the clinician is advised to keep abreast of changes. Furthermore, the question as to whether genetic factors or treatment response should primarily guide transplant decisions in CR1 is a subject of debate.

## HR cytogenetic features

A subset of *cytogenetic abnormalities* correlates with an increased risk of disease recurrence and induction failure, thereby negatively impacting survival [23]. The Medical Research Council)-AML group as well as the AML-BFM study group defined a monosomal karyotype, including monosomy -5, -7, del(5q), abnormal 3q, and 12p abnormalities as indicators of poor prognosis [19, 20, 24–26]. Monosomy 7 or del(7q) is associated with poor outcomes, but 5-year OS is highly variable based on other cytogenetic events, which is not addressed by most studies. 5-year OS for -7 can range from 5% with co-occurring -5/del(5q), inv(3), or +21, to 35% in patients without additional unfavorable cytogenetics [24, 25]. In contrast, deletion of 7q [del(7q)] shows a somewhat better prognosis, with a 5-year OS of 51%, and is considered more favorable than monosomy 7 [25], but variability dependent on co-occurring abnormalities is retained.

Similarly, monosomy 5 or del(5q) are associated with poor outcome with 5-year OS of 27% and 23%, respectively, where it has to be noted that the majority of cases harbor additional abnormalities and isolated -5/del5q is too rare to assign an individual prognostic impact. Of note, improved outcomes have been reported in patients with monosomy 7 or monosomy 5 when undergoing HSCT, but patients remain a poor risk group [23].

Abnormalities of chromosome 3q alone have not been reported to impact prognosis [19]. However, there is a strong association of -7/del(7q) and 3q abnormalities, and these patients belong to the group of highest risk, since the 5-year OS is as low as 5% for inv(3)/-7. In addition, AML with inv(3)(q21q26.2)/t(3;3)(q21;q26.2), which occurs in ~1% of newly diagnosed pediatric AML, has a very poor prognosis [27–30]. Survival of patients with abnormalities of 12p ranges from 22% to 35% [19, 20]. Overall, the sparsity of patients with these rare abnormalities and the heterogeneity of confounding factors included in the various studies make it difficult to precisely quantify their prognosis. Nevertheless, most patients with abnormalities of chromosome 5, 7, or 12p have to be regarded as HR and are likely to benefit from HSCT.

Some, but not all, cooperative study groups consider a complex karyotype (three or more unrelated chromosomal abnormalities in the absence of other class-defining recurring genetic abnormalities) to also confer an HR of relapse, with an event-free survival probability (EFS) of only 30% to 40% [19, 20].

11q23/*KMT2A* (formerly *MLL*) rearrangements *(KMT2A-*R*)*, which are highly prevalent in pediatric cohorts, accounting for 20-24% of AML cases, are associated with a heterogeneous outcome, depending on the fusion partner involved [19, 24, 31, 32]. In retrospective analyses, which include data from 756 patients with *KMT2A*-R AML, t(4;11)(q21;q23.3)/KMT2A::MLLT2, t(6;11)(q27;q23)/KMT2A::MLLT4, t(10;11)(p12;q23)/KMT2A::MLLT10, and t(10;11)(p11.2;q23)/KMT2A::ABI1 were associated with a dismal prognosis when treated with chemotherapy alone [31–35].

Yuen et al. reported a 5-year EFS of 49% and OS of 67% in pediatric AML patients with *11q23/KMT2A-R*, which were significantly worse compared to those of patients without such rearrangements. Among the subtypes, patients with t(6;11)(q27;q23)/KMT2A::MLLT4 had particularly poor outcomes, with a 5-year EFS of 13% and OS of 24%. Those with t(10;11)(p12;q23)/KMT2A:: MLLT10 showed a 5-year EFS of 23% and OS of 57% [36]. A retrospective study of >750 children with *KMT2A*-R treated by European and US cooperative groups found that t(4;11)(q21;q23), t(6;11)(q27;q23), t(10;11)(q12;q23), and t(10;11)(p11.2;q23) were associated with a 5-year EFS of only 10% to 40% [31]. To note, introducing Gemtuzumab Ozogamicin (GO) into treatment regimens and incorporating MRD improved prognosis for both HR and non-HR *KMT2A*-R patients [32, 34].

A number of rare chromosomal translocations are associated with a poor prognosis and are considered an indication for transplantation in CR1. These include the *FUS::ERG* translocation resulting from t(16;21)(p11;q22), which is associated with an extremely poor prognosis [37] and the t(9;22)/*BCR::ABL1* translocation [38–40]. Patients with FUS::ERG-positive AML are often primary refractory, or relapse quickly, and in Children’s Oncology Group (COG) clinical trials, 100% of transplanted patients succumbed to their disease [41]. The median survival time of patients with Philadelphia chromosome-positive AML was reported to be 7.5 months [42]. t(6;9)(p22;q34)/*DEK::NUP214*, often associated with FLT3 internal tandem duplication (FLT3/ITD) (~40% of cases), has a high RR with chemotherapy alone and is also considered an indication for an allograft in CR1 [43–45]. Data in both pediatric and adult populations, although obtained in small cohorts, show an OS higher than 50% for this subtype following HSCT in CR1 [43, 44].

Furthermore, cryptic gene fusions, including NUP98-rearrangements—t(5;11)(q35;p15)/*NUP98::NSD1*, t(11;12)(p15;p13)/*NUP98::KDM5A*—, inv16/*CBFA2T3::GLIS2* and t(7;12)(q36;p13)/*MNX1::ETV6* predict a poor outcome [46–50]. The 5-year OS for NUP98 fusions was 35% versus 64% in a reference group [51]. Specifically, NUP98-NSD1 patients had an OS of 36% and EFS of 17%, while NUP98-KDM5A patients had an OS of 30% and EFS of 25%. RR was also significantly higher: 64% for NUP98-NSD1 and 68% for NUP98-KDM5A, as compared to the reference group. Treatment response, measured by 5-year DFS post-induction, was lower in all NUP98 subtypes (27% vs. 52% in a reference group): NSD1 and KDM5A (28%) [51]. CBFA2T3-GLIS2 AML, an extremely aggressive AML subtype occurring in very young children, has a poor prognosis, with 5-year OS ranging from 14% to 42% and 5-year EFS ranging from 8% to 33% [52]. The patients with t(7;12) had a 3-year EFS of 24-43% [53]. These high failure rates highlight the need for new treatment modalities. An example is CBFA2T3-GLIS2 AML, where luveltamab tazevibulin, an antibody-drug conjugate (ADC), is currently being employed as a bridge to transplant and/or maintenance therapy (MT) post-transplantation (Supplementary Table 1). Finally, the t(8;16)(p11;p13)/*KAT6A::CREBBP* rearrangement has an important age-dependent impact on prognosis: in very young infants/neonates this translocation can be associated with spontaneous remission supporting a watch-and-wait strategy, whilst in older children the prognosis is poor and represents a criterion for HSCT in CR1 [54].

There is a very limited number of *monogenic HSCT classifiers*: only the internal tandem duplications in FLT3 (*FLT3-ITD*), which occur in ~10–20% of pediatric AML patients, either alone or with WT1 co-mutations, have proven to be of prognostic significance in de novo disease and at relapse [55–57]. In this context, patients with both NUP98 gene fusion co-expression and FLT3/ITD mutation have an EFS of only 13%, compared to 31% in patients with the FLT3/ITD mutation alone [58]. FLT3-ITD may not be HR in the presence of a low allelic ratio (AR < 0.5) or concomitant NPM1 mutations, but it can have an unfavorable prognostic value when associated with a high allelic ratio (HAR > 0.5) and without an NPM1 mutation [57, 59–63]. A COG study demonstrated that HAR FLT3/ITD mutation is associated with a 16% 4-year progression-free survival (PFS) rate and an 83% RR, which are significantly worse compared to those with FLT3 wild-type (FLT3/WT) [61]. However, it will be crucial to assess the prognostic value of FLT3-ITD AR when accounting for co-occurring mutations [57, 64, 65] and the significance of HAR is not as strong as initially found when considering the broader mutational landscape, but more comprehensive studies are needed. In a recent study, high FLT3-ITD AR retained its prognostic significance when accounting for NPM1, CEBPA, or WT1 status and patients with HAR did benefit from HSCT [57].

*AML with myelodysplasia-related changes* (MRC) is a subtype characterized by both blast count and genetic features. According to the 5th edition of the World Health Organization (WHO) Classification of Myeloid Neoplasms, this condition is now classified as “AML, myelodysplasia-related (AML-MR),” retaining the criterion of over 20% blasts, which distinguishes it from myelodysplastic syndrome (MDS). In the ITCC (International Therapeutic Classification of Cancer) system, the category of AML-MRC has been replaced by two distinct entities: AML with myelodysplasia-related cytogenetic abnormalities and AML with myelodysplasia-related gene mutations, neither requiring dysplastic features. Both the ITCC and WHO classifications include a complex karyotype as a defining feature of AML-MR or AML with myelodysplasia-related cytogenetic abnormalities [66].

Genetic features (chromosome abnormalities and genetic mutations) include unbalanced abnormalities such as del(5)/t(5q), -7, del(11q), del(12p)/t(12p), 13/del(13q), i(17q), del17p/t(17p), -idic(X)(q13). Additionally, mutations in genes such as *TP53, ASXL1*, *BCOR*, *EZH2*, *SF3B1*, *SRSF2*, *STAG2, U2AF1*, and *ZRSR2* are commonly observed [14, 66]. Patients with AML-MRC must undergo HSCT that can be preceded by a pre-transplant treatment, aimed at reducing the blast percentage prior to transplantation [67].

*Therapy-related (t)-AML* (t-AML) and *secondary AML* (s-AML) are indications for HSCT, irrespective if they arise from a germline mutation and/or are chemotherapy- or radiotherapy-related [68]. Gene mutations seen in t-AML and s-AML include activating mutations in tyrosine kinase RAS/BRAF pathways leading to an increase in cell proliferation, inactivating mutations in genes encoding hematopoietic transcription factors resulting in disrupted cell differentiation, and inactivating mutations in the tumor suppressor gene TP53. Currently, 10–20% of diagnosed AML and MDS cases are therapy-related, making t-AML the most common secondary malignancy in adults [69] and accounting for 1–3% of cases in children [68]. Retrospective studies have shown the challenges of determining the optimal preparative regimen [70]. Induction response rates and OS are poor, and children with t-AML have an HR of relapse and toxicity as a result of previous chemotherapy exposure for their initial disease. The choice of induction therapy must consider the cumulative drug dose—especially of anthracyclines (see below, section “Chasing CR: how to treat relapsed or secondary refractory pediatric AML?”)—and the often-impaired regenerative capacity of the bone marrow (BM). Due to the HR of additional toxicity, patients should proceed to HSCT after minimal induction chemotherapy, generally following 1 or 2 cycles of chemotherapy to induce remission [70–72]. Children achieving CR with full hematological recovery, CR with partial regeneration (CRp), or with no evidence of leukemia (NEL) have a considerable chance of being cured by HSCT. However, patients who fail to achieve morphological CR (<5% blasts) are unlikely to benefit from HSCT [70, 73].

At the time of diagnosis of t-MDS/AML, patients may present with a concomitant active neoplastic disorder [74], however, thorough results on HSCT for secondary hematologic malignancies in the context of an active primary solid tumor have not been reported in pediatric patients and will therefore not be further discussed in this review.

## Measurable residual disease monitoring as the “go to solution”

Some study groups place greater emphasis on MRD rather than cytogenetics or molecular biology when determining treatment options in first-line therapy [75].

The European Leukemia Net-MRD Working Party has provided guidelines for standardizing MRD assessment by either multiparametric flow cytometry (MFC-MRD) or molecular biology methods, including recommendations on the methodologies, time points for assessment, MRD thresholds, and definition of response [76, 77]. Commonly, the leukemia-associated aberrant immunophenotype (LAIP) and/or different-from-normal phenotype (DfN) are used for MRD assessment in children with AML (Fig. 2). However, molecular MRD monitoring by reverse-transcription quantitative PCR is often used in patients with FLT3-ITD, NPM1c, and fusion genes like RUNX1::RUNX1T1 and CBFB::MYH11 [76]. Molecular markers for MRD should be chosen based on their genetic stability in the leukemic clone [78].

Early assessment of MRD—during and after each course of induction, at the end-of-induction (EOI), and prior to consolidation therapy—helps in identifying patients most likely to benefit from therapy intensification, including HSCT [79, 80]. HR patients with EOI MRD > 1% showed a trend toward improved OS with consolidative HSCT compared to those without HSCT: 44% versus 23% [79]. In this study, MFC-MRD was applied as risk-stratification criteria together with genetic features to a cohort of 232 children consecutively enrolled in the AML02 multicenter trial [79]. MRD-positivity, defined as ≥0.1% of the mononuclear BM cells after induction 1, was associated with an unfavorable outcome in HR AML. Moreover, any MRD-positivity after induction 2 was predictive of an adverse outcome. This combined approach (MFC-MRD and genetic features) showed a 3-year EFS and OS of 63% and 71%, respectively. 80% (155 of 193) of patients achieved MRD of <0.1% after induction 2, and the cumulative incidence of relapse (CIR) for this group was 17%. MRD of ≥1% after induction 1 was the only significant independent adverse prognostic factor for both EFS and OS.

Moreover, the persistence of MFC-detectable MRD after EOI has been associated with inferior EFS and OS, as demonstrated in the Nordic Society of Pediatric Hematology and Oncology (NOPHO)-AML 2004 trial [81], and the COG AAML03P1 protocol [82]. In 2016, Tierens et al. retrospectively analyzed MFC-MRD prognostic impact (≥0.1% leukemic events were considered MRD-positive) at two different time points (day 15 of induction therapy and before consolidation therapy) in a cohort of 201 children enrolled in the NOPHO-AML 2004 trial [81]. In a multivariate analysis, only MFC-MRD-positivity before consolidation therapy was associated with an unfavorable outcome, with a strong impact both on EFS and OS. Recent findings further suggest a predictive role for combined blood (day 8) and BM (day 22) MRD [83].

European cooperative groups have also identified MFC-MRD 0.1% after EOI as an independent prognostic feature for relapse-free survival (RFS) and OS [84, 85]. In a retrospective study on the prognostic role of MFC-MRD in a cohort of 142 pediatric AML patients treated according to the Associazione Italiana di Ematologia e Oncologia Pediatrica (AIEOP)-AML 2002/01 trial [85], respective MRD levels (<0.1% vs. ≥0.1%) after induction 1 correlated with 8-year DFS (73% vs. 35%) and OS (82% vs. 51%). Similar results were observed for MRD levels after induction 2 (8-year DFS: 68% for MRD < 0.1% vs. 21% for MRD ≥ 0.1%; 8-year OS: 77% for MRD < 0.1% vs. 55% for MRD ≥ 0.1%). In a multivariate analysis, MRD ≥ 0.1% after induction 1 was associated with an adverse outcome.

These findings suggest that intensification of therapy through HSCT may improve outcomes in patients with a suboptimal treatment response at the EOI.

In recent years, several studies have explored real-time quantitative polymerase chain reaction (qPCR)-based molecular MRD as a predictor of relapse. However, the lack of standardized protocols, cut-offs, and time points—especially in pediatric settings—has limited its routine use before HSCT. An international retrospective (I)-BFM-AML study supports the clinical utility of qPCR-MRD in transplant management as a potential alternative to MFC [86]. Benetton et al. identified 2.1 × 10^−4^ as the most informative cut-off, distinguishing patients with low relapse risk (10.4%) and excellent OS (82.8%). A higher cut-off of 1 × 10^−2^ identified a subgroup with poor OS (<40%). These findings highlight molecular advances in improving sensitivity and specificity, making qPCR-MRD a potentially valuable complement to MFC assessment in guiding clinical decisions for HSCT in CR1, while its prognostic significance post-transplant remains unclear.

Altogether, recent meta-analyses have demonstrated that HSCT in CR1 improved OS and DFS with reduced RR compared to chemotherapy alone in 1448 HR pediatric AML patients treated between 1998 and 2017 [8]. Recommendations to proceed to HSCT in CR1 in these patients were based on multiple parameters, including unfavorable genetic alterations (see section “AML with myelodysplasia-related changes”), failure to achieve CR, or poor response to first-line treatment as measured by morphology and EOI MRD, which were based on each trial risk assessment [8]. Hence, most groups use a combination of genetic abnormalities and treatment response as detected by MFC-MRD to define patients as HR and offer them to HSCT. In particular, EOI MRD-positivity identifies additional patients at HR [84, 85, 87, 88], thereby guiding the selection of candidates for HSCT in CR1 [79].

Emerging methods for MRD monitoring include droplet digital PCR and next-generation sequencing (NGS), integrating genetic and transcriptomic analyses with circulating tumor DNA mirroring the genomic information from BM [89–91]. These technologies may provide the foundation for more precise HR definitions, and MRD-based risk stratification, potentially enabling treatment intensification of poor induction responders with HSCT in the future.

## How to deal with primary induction failure and primary refractory disease?

Treatment response, irrespective of genetic risk factors, is a strong predictor of outcome.

Achieving a deep MRD response is optimal prior to allograft [86]. However, patients with persistent blasts may still be curable by HSCT: patients with >30% AML blasts in the BM prior to HSCT have a leukemia-free survival (LFS) of 10%. An LFS of 10% in patients with a disease that is invariably fatal without HSCT may be acceptable to most families [92].

**Fig. 3:**
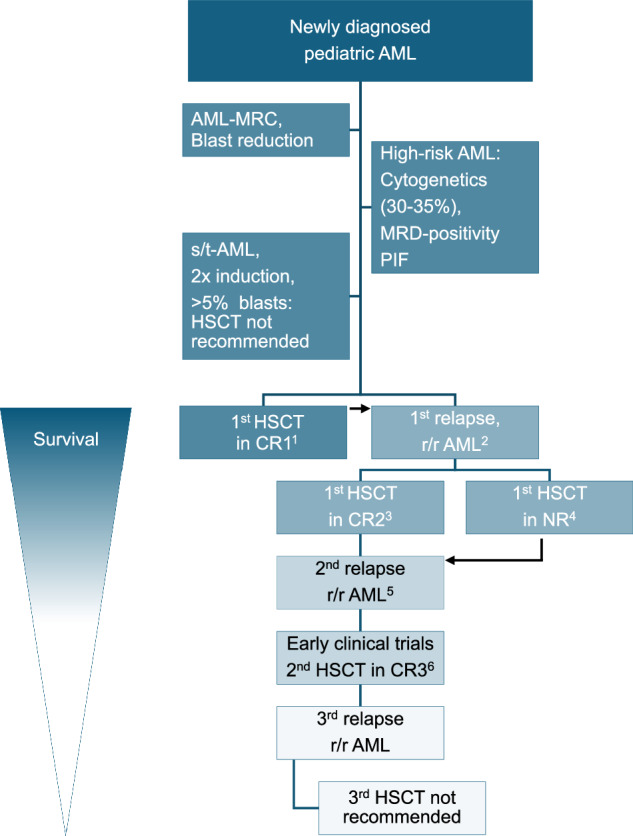
To transplant or not to transplant in pediatric AML? Retrospective analyses have demonstrated that HSCT in CR1 improves OS with reduced RR in high-risk and r/r pediatric AML patients. The prognostic significance of NR or MRD-positivity before HSCT, as well as the association of subsequent HSCT with poorer survival outcomes, has been confirmed by various study groups. Numeric details are provided below: ^1^AML-BFM (2010-2012): 5-year pOS: 76% [183]; AIEOP-2002/01: 8-year pOS: 74% [205]. ^2^AML-BFM (2011-2012): CIR: 25.1% (SE 3.9), NR: 12.3% (SE 2.8) [183]; AIEOP-2002/01: CIR: 17% [205]. ^3^AML-BFM 2004/2012 and AML-BFM registry 2012: 5-year pOS 54.5%, SE = 4.4; COG (AAML0531 and AAML1031) (2013–2017): 5-year pOS 40%; 5-year pOS 24%, MRD^+^_,_ COG: 5-year pOS 41%, MRD^−^ [106]. ^4^BFM 2004/2012 and AML-BFM registry 2012: NR patients: 5-year pOS 26.7%, SE = 9.0 [106]. ^5^AML-SCT-BFM: CR1/CR2: 4-year pOS and pEFS 61 and 70%, CIR 22%, NRM 15% [107]. ^6^2-year pLFS: CR 33%, NR 19%, 8-year pLFS: CR 24%, NR, 10% [206]. 4-year pOS w HSCT: 31% w/o 3%, CIR and NRM at 4 years: 45% and 22% [177]. *Consider maintenance therapy. AML acute myeloid leukemia, AML-MRC AML-myelodysplasia-related changes, PIF primary induction failure, s/t-AML secondary/therapy-related AML, r/r AML relapsed/refractory AML, MRD measurable residual disease, CR complete remission, NR no response, BFM Berlin–Frankfurt–Münster Study Group, COG Children’s Oncology Group, HSCT hematopoietic stem cell transplant, OS overall survival, RR relapse rate, CIR cumulative incidence of relapse, SE standard error, LFS leukemia-free survival, NRM non-relapse mortality.

For patients with primary induction failure (PIF) or refractory disease, the prognosis is poor without HSCT [93–95]. Patients with PIF may benefit from intensifying therapy and/or from novel therapeutic approaches, if available, to achieve morphological remission prior to HSCT [96, 97]. Achieving blast reduction is crucial for optimizing outcomes, as discussed in the next section and shown in Fig. 3.

## Chasing CR: How to treat relapsed or secondary refractory pediatric AML?

Approximately 30–40% of patients with de novo childhood AML experience leukemia relapse. These patients should be considered for HSCT in the second CR (CR2) [98–101]. Achieving CR2 in relapsed or secondary refractory patients is essential for optimizing post-HSCT outcomes. Several key factors must be carefully considered for increasing the likelihood of achieving CR2. These include the site of relapse, the time from the initial diagnosis to relapse, the patient’s response to reinduction therapy, and the cumulative anthracycline dose received (Table 1). Additionally, the availability of novel chemotherapeutic agents and the immunophenotype or presence of specific mutations/cytogenetics, which may identify appropriate targeted therapies, are important considerations in choosing effective strategies for these patients (Table 1 and Supplementary Table 1) [98, 102–105].

**Table 1:** Five prognostic benefits of HSCT for relapsed/refractory pediatric AML.

Key factor	Predictive markers for outcome	References
Site of relapse	• Extramedullary disease (EMD) did not affect transplant outcomes • Controlling central nervous system (CNS) EMD is essential before performing HSCT	[194–196]
Time from remission to relapse	• Relapse within 1 year (early relapse) is associated with poor long-term survival	[106]
Response to remission induction therapy	• Efforts should be made to achieve significant treatment responses before HSCT: o Cumulative anthracycline exposure of <450 mg/m^2^: Two cycles of chemotherapy o Cumulative anthracycline exposure of ≥450 mg/m^2^: Alternative relapse regimens and novel drugs including immunotherapeutics	[55, 197–201], Supplementary Table 1
Remission induction therapy: Current concept	• Intensive reinduction with one or two courses followed by HSCT once in CR or in Aplasia	[23]
Remission induction therapy: Novel drugs	• The transfer to an early clinical trial or large randomized trials should be considered if available	[97, 200, 202–204], Supplementary Table 1

Outcomes for HSCT in children with relapsed AML have improved over time: In the COG cohorts AAML0531 and AAML1031, the 5-year pOS was 33% and 37%, respectively. For patients relapsing between 2013 and 2017, the 5-year pOS was 40% [106]. Similarly, the BFM study group reported an improvement in 5-year OS from 39% in 2009–2013 to 49% in 2013–2017 [106]. Patients in CR2 treated on the AML-SCT-BFM 2007 trial, which focused on conditioning regimens, had a 4-year EFS of 46% and a RR of 27% [107]. Next, a multicenter retrospective analysis of 343 children with acute leukemia (AL) treated between 2010 and 2015 demonstrated that HLA-haploidentical related α/β T-cell- and B-cell-depleted transplantation (α/β-haploidentical-HSCT) was equally effective as matched unrelated donor (MUD) HSCT [108]. This finding supports the use of α/β-haploidentical-HSCT for children with AL who either lack a matched donor or require urgent transplantation without sufficient time to identify a suitable MUD. The 5-year probability of LFS for either MUD, mismatched unrelated donor (MMUD), or α/β-haploidentical-HSCT transplantations was 67%, 55%, and 62%, respectively, with all patients being in morphological remission and receiving myeloablative conditioning (MAC) regimens. Notably, compared to MMUD-HSCT, children treated with α/β-haploidentical-HSCT had a lower incidence of grades II–IV acute and chronic graft-versus-host-disease (aGvHD/cGvHD), as well as a lower cumulative incidence (CI) of non-relapse mortality (NRM). The probability of cGvHD-free/relapse-free (GRFS) was 34% after MMUD-HSCT versus 58% after α/β-haploidentical-HSCT, while NRM rates were 28% and 9%, respectively [108]. More recently, the European Society for Blood and Marrow Transplantation reported a matched-pairs analysis of children with AML in CR1 or CR2 who underwent either MUD HSCT with anti-thymocyte globulin (ATG) (*N* = 253) or unmanipulated haploidentical-HSCT with post-transplant cyclophosphamide (PT-CY) (*N* = 95) following MAC conditioning between 2011 and 2021 [109]. Despite a higher incidence of grade III-IV aGVHD in the haploidentical group, no significant differences were observed in 2-year OS (78% vs. 71%), LFS (72 vs. 69%), CI of relapse (19% vs. 19%) NRM (8% vs. 11%) and GRFS (60% vs. 54%) between the MUD and haploidentical-HSCT groups, respectively. This study indicates that haploidentical-HSCT with PT-CY is a suitable alternative for children with AML who lack a matched donor [109].

Obtaining deep CR in relapsed or primary refractory patients is crucial to improve outcomes, as reported in a prospective study in 123 children who proceeded to HSCT in CR with a significantly better DFS in MRD-negative compared to MRD-positive patients [110]. In independent cooperative group studies conducted by COG, 765 of 852 (90%) patients were monitored by flow cytometry for residual disease (RD) [106]. The 5-year OS probability following relapse for patients who were RD-positive at the EOI was 24% (*n* = 222) and 41% for those who were RD-negative (*n* = 543, *p* < 0.001).

According to Wang et al., the OS rates of 37 pediatric AML patients treated with matched family donor (MFD) HSCT in CR1 after induction therapy with the Chinese Children’s Leukemia Group–AML2015 regimen were 89%, 75%, and 75%, while the RRs were 11%, 24%, and 33% at 1, 3, and 5 years post-HSCT, respectively [111]. 14 patients (37%) received donor lymphocyte infusions (DLI) due to positive MRD post-transplantation, and relapse was recorded in 9 patients (3–46 months post-HSCT), with a second HSCT performed in 5 patients. Notably, Homoharringtonine-based induction therapy was superior to Etoposide-based induction therapy, which highlights the need for more effective therapeutic strategies for patients who relapse after HSCT. In line with this, Tierens et al. reported on a phase III study using an intensified, response-guided induction regimen containing liposomal Daunorubicin (DNX) or Mitoxantrone, along with MRD-based risk stratification on day 22 after induction 1 and again after induction 2 [112]. Patients with poor induction response (Mitoxantrone had a superior anti-leukemic effect) were treated with HSCT. 5-year EFS and OS rates were 77% and 83%, respectively, for HR patients with HSCT (85%) [112]. Intensification of induction, risk stratification on the basis of treatment response, and treatment intensification with HSCT in HR patients led to improved HSCT outcomes.

Despite the major benefit of achieving CR, there is evidence supporting the use of a consolidative HSCT in the absence of CR in subsets of patients: the BFM study group reported an OS rate of 27% at 5 years in children with ≥5% residual leukemic blasts after two courses of reinduction with DNX + Fludarabin + Cytarabin (DNX-FLA ± FLA), suggesting that HSCT has a role post-relapse even in the absence of a morphological CR [106]. However, advanced disease at HSCT continues to be associated with poorer survival outcomes in children [113, 114], and MRD remains a strong prognostic factor prior to HSCT, significantly influencing outcomes [115].

**Fig. 4:**
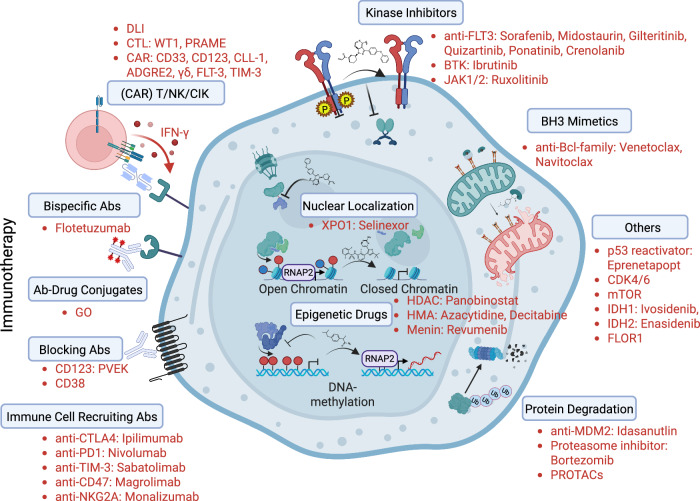
Overview of treatment targets currently available or under development in acute myeloid leukemia. Immunotherapy initiatives include *ADC* antibody (Abs)-drug conjugates, bispecific Abs, *mAbs* monoclonal Abs, *CI* checkpoint inhibitors, and cellular therapies (*DLI* donor lymphocyte infusion, *CTL* cytotoxic T lymphocytes, *CAR* chimeric antigen receptor approaches, *NK* natural killer, *CIK* cytokine-induced killer). Targeted therapies are summarized as *RTK* receptor tyrosine kinase inhibitors, *BH3 mimetics* selective small-molecule B-cell lymphoma 2 (Bcl-2) Homology 3, and inhibitors involved in protein degradation. WT1 Wilms tumor protein, PRAME preferentially expressed antigen in melanoma, ADGRE2 adhesion G protein-coupled receptor E2, FLT3 FMS‐like tyrosine kinase 3, TIM-3 T cell immunoglobulin and mucin-domain containing-3, GO gemtuzumab ozogamicin, PVEK pivekimab sunirine, CTLA-4 cytotoxic T-lymphocyte-associated protein 4, PD-1 programmed cell death protein 1, XPO1 exportin 1, HDACs histone deacetylases, HMA DNA hypomethylating agents, BTK Bruton’s tyrosine kinase, JAK Janus kinase, CDK cyclin-dependent kinase, mTOR mammalian target of rapamycin, IDH isocitrate dehydrogenase, FOLR1 folate receptor alpha, MDM2 mouse double minute 2 homolog, PROTAC proteolysis targeting chimera.

State-of-the-art multimodal chemotherapy and targeted therapies offer the potential to achieve CR prior to HSCT in relapsed AML patients. Ongoing efforts for improvement are highlighted by the multitude of studies in this pediatric cohort (Supplementary Table 1). Importantly, new compounds currently under development may offer more personalized therapies to these patients; however, more trials focusing on children are still urgently required (Fig. 4).

## Bridging to HSCT in the short-term

Whilst achieving CR is important prior to HSCT, delaying HSCT is a case of concern in the relapsed/refractory (r/r) disease setting. Reasons for postponing HSCT may include an unexpected delay in donor availability, e.g., due to temporary inability to donate or inability to undergo anesthesia or mobilization of peripheral stem cells, withdrawal of consent to donate, limited number of collection centers, etc. In these circumstances, it is crucial to maintain CR, and short-term bridging therapy is advised. Currently, the choice of pre-transplant bridging therapies includes consolidation with high-dose (HD)-Ara-C monotherapy, GO (although the risk of liver veno-occlusive disease cannot be scotomized), Venetoclax/Azacytidine (VEN/AZA) or low-intensity consolidation with LD-Ara-C and Thioguanine, depending on disease dynamics and the time span to be bridged. In the absence of relevant studies, individualized patient management should be considered, particularly for heavily chemotherapy pretreated patients with persistent disease prior to HSCT (Fig. 4). This includes targeted therapies and, where applicable and/or available in the pre- and/or post-transplant setting (in case of a prior HSCT) [116], cellular immunotherapies such as DLI, cytotoxic T lymphocytes (CTL), natural killer (NK) cells, cytokine-induced killer (CIK) cells or, in the future, their chimeric antigen receptor (CAR) modified counterparts, including CAR-T, CAR-NK, and CAR-CIK cells.

## What do we need to consider when choosing the donor stem cells? A debate on the best stem cell donor and stem cell source

The choice of donor is complicated by limited clinical data which are often outdated and involve small AML patient cohorts [108]. When discussing graft selection, two interconnected aspects must be addressed simultaneously: **HLA matching** and the choice of stem cell **source**.

HLA-identical sibling donors have historically been the preferred donor, balancing relapse occurrence and the risk of developing GvHD. Currently, the outcome of patients transplanted from an HLA-identical sibling, or a fully matched unrelated volunteer (URD) is comparable [117].

**Fig. 5:**
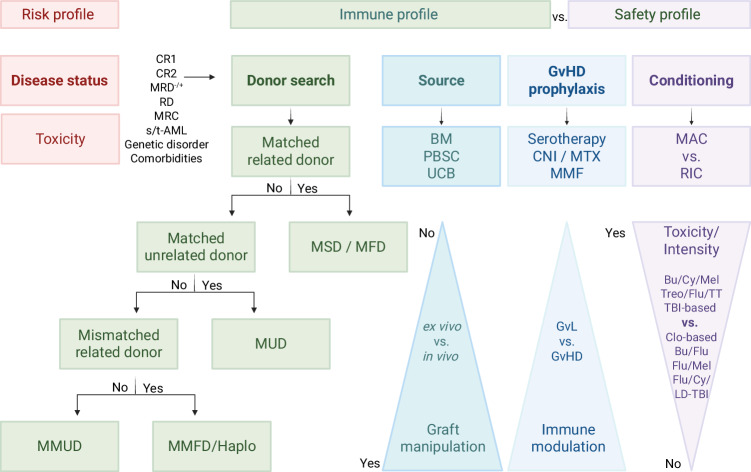
A Guide to personalized therapy: HSCT in pediatric AML. The choice of donor and conditioning regimes for patients with an indication for allogeneic HSCT for pAML. Conditioning regimes are considered “reduced intensity/toxicity (RIC)” if the dose of Busulfan is less than 8 mg/kg PO or IV, Melphalan is less than 150 mg/m², total body irradiation (TBI) dose is ≤500 as a single dose, or 800 cGy administered as fractionated doses. Other regimens are considered myeloablative (MAC). pAML pediatric acute myeloid leukemia, HR high-risk, AML-MRC AML-myelodysplasia-related changes, s/t-AML secondary/therapy-related AML, r/r AML relapsed/refractory AML, MRD measurable residual disease, RD residual disease, CR complete remission, HSCT hematopoietic stem cell transplant, HLA human leukocyte antigen, MSD matched sibling donor (second choice in case of a minor child), MFD matched family donor, MUD matched unrelated donor, MMFD mismatched family donor, MMUD mismatched unrelated donor, BM bone marrow, PBSC peripheral blood stem cells, UCB umbilical cord blood, GvHD graft-versus-host disease, GvL graft-versus leukemia, CNI calcineurin inhibitor, MTX methotrexate, MMF mycophenolate mofetil, MAC myeloablative conditioning, RIC reduced intensity conditioning, Bu Busulfan, Cy Cyclophosphamide, Mel Melphalan, Treo Treosulfan, Flu Fludarabine, TT Thiotepa, (LD)-TBI (low dose) total body irradiation, Clo Clofarabine.

In the absence of an HLA-MFD, an immediate search for a MUD should be initiated at diagnosis for patients with AML-MRC, t-AML, s-AML, r/r AML, or HR AML (Figs. 3 and 5). Several studies have found that the killer-cell immunoglobulin-like receptor (KIR) alloreactivity in the donor/recipient pair is associated with AML outcome [118–121]. However, recent data from children with acute lymphoblastic leukemia (ALL; *n* = 372) or AML (*n* = 344) who received well-matched, T cell–replete, or in vivo T cell–depleted, URD transplantation, and who were reported to the Center for International Blood and Marrow Transplant Research (CIBMTR) between 2005 and 2016, showed that KIR ligand mismatch, KIR gene content (Cen B), KIR2DS1 mismatching, and Cen B/telomeric A were not significantly associated with relapse or DFS in the AML subgroup undergoing well-matched URD transplantations [122].

Additionally, in the absence of a matched donor for HR AML, the use of mismatched/haploidentical family donors (MMFD), MMUD, and unrelated umbilical cord blood (UCB), HSCT should be considered. In this context, Fierro-Pineda et al. reported similar feasibility, safety and efficacy rates for matched donor and haploidentical-HSCT allografts in a small cohort of 15 patients with AML or MDS [123]. Wang et al. and Ciurea et al. both reported an equivalent DFS of 75% for haploidentical versus matched sibling (MSD) HSCT in CR1 in larger cohorts of pediatric and adult patients with intermediate- and HR AML [124, 125]. Moreover, a retrospective review of children with AML in CR1 reported a 75% OS for haploidentical recipients [126]. However, more confirmatory results are urgently needed in the pediatric AML setting. Clear recommendations on which HLA-haploidentical-HSCT approach should be preferred cannot yet be made, although promising results have recently been published with the use of α/β T cell- and B-cell-depleted allografts [108, 109, 127].

The second consideration of graft selection is the stem cell *source*. Both unmanipulated BM and peripheral blood stem cells (PBSC) are generally acceptable options (Fig. 5). However, caution on the use of PBSC is advised in adolescents and in transplants from female donors to male recipients due to an increased risk of GvHD. In cases of mismatched HSCT, the use of unmanipulated PBSC should be approached with even greater caution, and ex vivo or in vivo T cell depletion (TCD) may be necessary to mitigate the GvHD risk [128].

While UCB is less frequently used in the last years, it remains a potential stem cell source for HSCT [129]. Recipients of UCB-HSCT (UCBT) may be at higher risk for early and increased TRM, delayed platelet and neutrophil engraftment, and slower immune reconstitution compared with recipients of BM and PBSC grafts [130]. However, UCB recipients have a reduced risk of cGvHD and the limitation of delayed neutrophil engraftment has improved in more recent experience [131–134]. Recent OS and EFS outcomes of single (s)UCBT in children with HR and refractory AML were 71% (95%CI 62%-77%) and 72% (95%CI 64%-78%), respectively [134]. The guidelines from the National Marrow Donor Program and the CIBMTR recommend the following criteria for sUCBT: a minimum of 8/8 high-resolution HLA typing (HLA-A, HLA-B, HLA-C, and HLA-DRB1), ≥4/6 typing for HLA-A and HLA-B antigens, high-resolution HLA-DRB1 typing, ≥4/8 high-resolution typing, a total nucleated cell (TNC) count ≥2.5 × 10^7^/kg, and a CD34^+^ cell count ≥1.5 × 10^5^/kg [135]. HLA-C and KIR combinations have been reported to significantly impact RFS in UCBTs for AML [136]. Patients receiving a 7/8 ABCDR-matched graft with a single HLA-C mismatch experienced significantly poorer RFS than 8/8 matched UCBTs (*P* = 0.04). The 5-year cumulative recurrence rates of CR1, CR2, and non-responder (NR) groups were 5%, 19%, and 30%. This suggests that, for pediatric patients with AML, UCBT may be a suitable alternative option.

In this respect, outcome comparisons of MFD, MUD, and unrelated UCBT in AML showed no difference in RR or LFS but improved cGvHD-free survival [137] and, in particular, EFS [138] for unrelated UCBT. Horgan et al. suggested that UCBT without serotherapy could be the optimal transplant option for children with MRD-positive myeloid malignancy [138]. Furthermore, current evidence from a large multicenter retrospective study involving 316 recipients of MSD, MUD, UCB, and double UCB transplants demonstrated no significant differences in RR, LFS, or NRM based on the stem cell source.

The choice between BM or PBSC is influenced by collection logistics, and extreme weight differences between donor and recipient, which could limit the ability to obtain adequate numbers of hematopoietic stem cells from BM. PBSC grafts compared with BM are associated with higher rates of cGvHD. If the use of unmanipulated PBSC is necessary, PT-Cy can be employed as an effective in vivo T cell-depletion strategy to mitigate the risk of cGvHD. PBSC grafts are generally not recommended as the preferred stem cell source, particularly from unrelated donor transplants [139–141].

Despite significant challenges, more prospective studies to advance our understanding of the best suitable stem cell sources, the optimal number of cells to infuse, and individualized donor-recipient matching [142] would be desirable and may help refine GvHD prophylaxis and tailor allograft approaches to improve outcomes in allogeneic HSCT.

## How is optimal HSCT performed in terms of conditioning?

After donor selection, determining the appropriate conditioning regimen is another critical step. Conditioning regimes are separated into “reduced intensity/toxicity (RIC)” with Busulfan (Bu) dosing of <8 mg/kg PO or IV equivalent, Melphalan (Mel) dosing <150 mg/m², total body irradiation (TBI) dose of ≤500 cGy as a single dose, or 800 cGy administered as fractionated doses [143]. In contrast, regimens are considered MAC for any higher dosing. MAC is generally recommended for children with AML. The choice of conditioning regimen should be carefully balanced taking into consideration potential toxicities, the individual patient’s prior treatments and current performance status, and any existing infectious complications at the time of transplantation.

A large retrospective CIBMTR study found no difference in OS or LFS between TBI-based and non-TBI-based MAC regimens [144]. In view of the risk of increased late effects associated with TBI, MAC conditioning with Bu, Cyclophosphamide (Cy) and Mel may be used for patients transplanted in CR1 and CR2 [139]. It is recommended to use weight- and a pharmacokinetically-adjusted dose of Bu to minimize toxicity and maximize efficacy. In the AML-SCT-BFM 2007 trial, the 4-year EFS, OS, CIR, and TRM rates were 61%, 70%, 22%, and 15%, respectively [107]. Notably, TRM varied significantly by age: it was 9% (SE 3%) in children under 12 years, but increased to 31% (SE 9%) in children aged 12 years and older [107]. However, contemporary HSCT regimens and improved HLA typing have largely mitigated these age-related differences, making HSCT with either Bu or Treosulfan (Treo) conditioning safer for older pediatric and young adults (AYA) aged 16-20 [145].

In response to these outcomes, the current trial being conducted by AIEOP/BFM is randomizing patients to receive either Bu/Cy/Mel or Treo, Fludarabine (Flu), and Thiotepa (Thio). This trial could hopefully help determine the optimal MAC regimen, not limited to patients with AML older than 12 years. Additionally, the ongoing prospective, randomized SCRIPT-AML study by the NOPHO-DBH (Dutch-Belgian-Hong Kong) consortium is testing the hypothesis that increasing Bu exposure to achieve a cumulative area under the curve of 90 mg*h/L and replacing alkylating agents (Cy, Mel) with antimetabolites (Clofarabine(Clo), Flu) could improve outcomes.

Whilst MAC is the regimen of choice for most patients, a subset of patients—particularly those with underlying genetic disorders—who are at risk of significantly higher TRM rates should be considered for RIC regimens. This special category includes children with Down syndrome, MDS/leukemia predisposition syndromes, children with an inherited bone marrow failure syndrome such as Fanconi anemia, Schwachman-Diamond syndrome, congenital amegakaryocytic thrombocytopenia, Diamond-Blackfan anemia, dyskeratosis congenita, and severe congenital neutropenia.

Similarly, children with significant comorbidities or those undergoing a second HSCT may also benefit from RIC instead of MAC conditioning. RIC regimens include combinations such as Bu/Flu, Flu/Mel, or regimens incorporating Clo or Treo [146]. In CD33^+^ pediatric AML patients in CR1/CR2, the pOS and EFS at 5 years following RIC HSCT (Bu/Flu) and GO consolidation were 61% and 78%, respectively [147]. Children with poorly responding primary disease or relapse who underwent early HSCT after a cytoreductive regimen with Flu, Amsacrine, and Cy, followed by RIC conditioning and prophylactic DLI, had 4-year EFS and OS rates of 49% and 53%, respectively. CIR was 38%, and TRM was 11% [107]. These findings support the benefit of consolidative HSCT in subsets of patients, even in the absence of CR, as previously reported in the section *“*Chasing CR: how to treat relapsed or secondary refractory pediatric AML?*”*.

Two retrospective studies of 141 and 34 children with AML [144, 148] found no significant difference between RIC and MAC in terms of OS, RR, and TRM. However, ongoing improvements in HSCT procedures, leading to declining TRM rates, may shift this balance. When managed carefully, MAC conditioning may improve long-term survival, even in heavily pretreated patients and those at HR for relapse, such as patients with refractory AML and t-AML.

## Janus’ face: graft-versus-leukemia (GvL) and GvHD

While the donor immune cell-mediated anti-leukemia effect is essential to a favorable HSCT outcome, acute and chronic GvHD are major contributors to mortality and morbidity, and the benefit of GvL must be balanced against the risks of GvHD (Fig. 5). This is particularly important in the presence of HLA disparity between donor and recipient and the use of PBSCs. Therefore, ex vivo TCD of the graft, such as the α/β TCD, is commonly used in pediatric patients undergoing transplantation from an HLA-haploidentical donor. As noted in paragraph 4, α/β-haploidentical-HSCT can be handled successfully and is a promising option for patients with no other graft available [108, 109].

In vivo, T-cell depleting serotherapy is administered pre-transplant to patients receiving grafts from MUD, MMFD, or 5–6/8 matched UCB, but is not typically used for those transplanted from an MFD or receiving a 6–8/8 matched UCB [138].

All patients given an unmanipulated graft usually receive immunosuppression (IS) with calcineurin inhibitors (CNI) for GvHD prevention starting prior to stem cell infusion. Patients receiving grafts from an MMD or PBSC, or unrelated UCB, should receive additional prophylaxis with either MMF (mycophenolate mofetil) or short-course Methotrexate (MTX). Short-course MTX is used for in vivo depletion of proliferating alloreactive T cells following HSCT in this setting. Patients who receive unmanipulated BM from a haploidentical parent can also be successfully managed with an in vivo TCD/modulation approach using PT-Cy.

In the absence of GvHD, MMF can be stopped at day 28 post-transplant, and CNI tapered over 4–6 weeks from day 60 (MFD), day 100 (MUD), or earlier if mixed chimerism or MRD is detected. This enhances the benefits of GvL (see section “Losing a battle is not losing the war: maintenance therapy post-transplant and second HSCT”). Optimization of anti-infective and supportive care treatment that does not disturb the gastrointestinal microbiome (GM)—thereby decreasing toxicity, relapse, and GvHD rates—should be considered in future patient management [149, 150].

As GM alterations are linked to leukemogenesis and treatment-related complications, especially during HSCT, various approaches can be used to modulate GM in children, including nutritional interventions, fecal microbiota transplantation, and prebiotics [151, 152]. While there is strong scientific rationale and emerging clinical interest, caution is needed when using probiotics or microbial agents mentioned above. One of the most effective ways to influence GM is by modulating antibiotic use with strategies such as narrow-spectrum antibiotics and optimizing treatment timing and duration. These approaches should be explored in future pediatric clinical studies.

## Losing a battle is not losing the war: maintenance therapy post-transplant and second HSCT

From 10% to 60% of patients relapse within the first year of HSCT depending on risk criteria as reported by Shahn et al. [153, 154]. A number of options have the potential to maintain remission post-HSCT (Figs. 3 and 4). These include withdrawal of immunosuppression, DLI, and—if available and applicable—CTLs, NK cells, CIK cells, or, in the future, CAR-T, CAR-NK, and CAR-CIK immune cell therapy. These later immunotherapies are specific/targeted therapies compared to bulk population DLI but are not yet available to most patients [155–164]. Hypomethylating agents (HMAs) (Decitabine and Azacytidine), alone or combined with DLI or rhG-CSF [165, 166], tyrosine kinase inhibitors (TKIs) (for FLT3-mutated AMLs) [167] and Venetoclax are frequently used for managing leukemia relapse after HSCT. TKI maintenance after HSCT is a promising treatment option for HR AML, leading to long-term remission with minimal side effects. For patients with FLT3-positive AML who initially received Midostaurin, a switch to Sorafenib should be considered after transplantation. Sorafenib is, to date, the only inhibitor that demonstrated efficacy in improving both PFS and OS as post-HSCT MT [168–171]. However, due to their improved safety profile and higher efficacy, second-generation FLT3 inhibitors such as Quizartinib or Gilteritinib can be used post-transplant, if available. Levis et al. showed that Gilteritinib was beneficial as post-HSCT maintenance in adults with FLT3-ITD AML who had detectable MRD, resulting in a significant RFS benefit, particularly in this MRD-positive subgroup [172]. However, the timing and duration of treatment with TKIs after HSCT have not yet been determined for children. In a retrospective study, 15 pediatric patients were treated with Sorafenib given for a median of 100 days post-HSCT and extended for a period of 18 months in some patients [173]. The addition of the FLT3 inhibitor Quizartinib to pre-transplant chemotherapy, in combination with post-transplant maintenance, will be studied in FLT3^+^ pediatric patients within the CHIP-AML protocol as part of a linked “Quizartinib” trial (recently approved and currently recruiting). Several targeted agents are being investigated in clinical trials (see Supplementary Table 1), with more in development for patients who relapse after a second HSCT and face a poor prognosis. However, there are insufficient data to clearly recommend which post-HSCT targeted-/immune-therapy should be used, and the decision is based on physician choice and drug availability when participation in a clinical trial is not possible.

A second HSCT may be considered in patients who have relapsed post-HSCT, and have achieved a significant reduction in blast count with reinduction chemotherapy (as defined above in the section “Chasing CR: how to treat relapsed or secondary refractory pediatric AML?”. and shown in Fig. 3), and have an acceptable performance status.

Retrospective studies on children who underwent a second HSCT have demonstrated a long-term DFS ranging between 10% and 50% [174–176]. However, the 5-year OS was only 15% [55], when patients with a poor response to reinduction therapy and an early relapse (<1 year) are included. Patients who relapsed >1 year after the first HSCT and who had a good response to reinduction chemotherapy had a better survival rate of 24–35% [176]. These findings were provided by the following studies: in a large cohort of 122 patients, 4-year OS, NRM, and CIR of 31%, 22%, and 45% were reported after the second HSCT [177]. In a smaller cohort of 46 children, a 5-year OS of 41.7% was reported, which increased with an inter-HSCT interval of >2 years (63% vs. 27%; *p* = 0.01) [175]. Furthermore, in a multicenter national analysis of mismatched T-repleted UCB, an impressive 2-year EFS of 69% was noted in a cohort of r/r AML patients with a previous history of HSCT (*n* = 24) [138].

Consequently, achieving CR following reinduction therapy is critical. Additionally, post-transplantation MT could be considered to maintain remission for more than 12 months after HSCT and to avoid inter-HSCT intervals of less than 6 months to reduce the risk of relapse and toxicity [174–176]. Conditioning for a second HSCT must be selected carefully based on the patient’s prior history and comorbidities. If a third complete remission can be achieved after a second HSCT, a 5-year OS of 40% has been reported [56]. No child or AYA with multiple relapsed AML has ever been reported to survive after a third HSCT (Fig. 3).

## Concluding remarks

This review highlights the current approach to HSCT in AML achieved through consecutive improvements of several study groups [6, 35, 106, 107, 178–191]. The internationally agreed principles for diagnosis, risk stratification, treatment response, and pre- and post-transplant management provide guidelines and recommendations for the treatment of AML patients with r/r or HR disease in CR1, both currently and in the future [5, 6, 192, 193].

Although survival rates have gradually improved in recent decades, there is still an urgent need for more effective and less toxic induction therapies and conditioning regimens for patients undergoing HSCT, in order to minimize both acute and late toxicity, and thereby further extend the use of HSCT as a curative approach in pediatric AML. Incorporating molecular disease characterization (epigenomic, genomic, transcriptomic) and MRD monitoring—including NGS—will continue to refine risk stratification and therapeutic decisions in CR1 and relapsed disease, and highlight opportunities for studies aiming to define timing of HSCT, optimal remission status before HSCT, clinical parameter-orientated donor selection and conditioning, and GvHD regimens, towards a more individualized patient management including the use of targeted therapies and cellular immunotherapy.

## Supplementary information

Supplementary Table 1
