# Supplementary material for: The hallmarks of hematopoietic stem cell transplantation for pediatric acute myeloid leukemia
Source: Leukemia. 2025 Jul 9;39(10):2313–28. doi: 10.1038/s41375-025-02685-5 (PMC12463678; doi:10.1038/s41375-025-02685-5)
Supplement: Supplementary file 1 — Supplementary Table 1 [file 41375_2025_2685_MOESM1_ESM.docx]

**Table 1. Selected early clinical trials for relapsed/refractory pediatric AML**

**(Supplement)**

| Study population | Target | Targeted therapy | Combinatoriall therapy | Sponsor/Trial | NCT/  EudraCT | Study design/Outcome |
| --- | --- | --- | --- | --- | --- | --- |
| Relapsed/refractory AML, CD33^+^, ≤21 years | CD33 | GO | Liposomal  Daunorubicin  and Cytarabine | MDACC | NCT04915612 |  |
| Relapsed/refractory AML, CD33^+/-^ | CD33 | GO | FLAG with GO with or without Venetoclax | International Leukemia & Lymphoma Society PedAL/EUpAL consortium APAL2020D | NCT05183035, EudraCT 2021-003212-11 | Randomized phase III |
| Relapsed/refractory AML, .≥1 and  ≤21 years | Liposomal preparation of Daunorubicin and Cytarabine | CPX-351 | Cycle 1:  CPX-351  Cycle 2:  FLAG | Jazz Pharmaceuticals (Palo Alto, California)  COG, AAML1421, first-in-child phase I study | NCT01943862 | Overall response rate: 81% in 37 treated patients of whom 29 received HSCT[207] |
| Relapsed/refractory AML, ≤19 years | Structural hybrid of Fludarabine and Cladribine | Clofarabine | Clofarabine with FLA + DNX | Innovative Therapies for Children with Cancer (ITCC) Consortium/BFM trial, ITCC 020 phase Ib | EUDRA-CT 2009-009457-13; Dutch Trial Registry number 1880 | Overall response rate: 68%  EFS 35 (FU, 1-y), OS 50% (1-y)[208] |
| Relapsed/refractory AML, .≥1 and  ≤21 years | Structural hybrid of Fludarabine and Cladribine | Clofarabine | Clofarabine and Cytarabine | COG AAML0523 phase II study | NCT00372619 | 3-year OS of 46%,  bridge to HSCT[209] |
| Relapsed/refractory acute leukemia | Proteasome inhibitor | Bortezomib | Proteasome inhibitors:  Bortezomib and either high-dose Cytarabine and Etoposide or low-dose Cytarabine and Idarubicin | COG AALL07P1 phase II/pilot trial | NCT00666588 | CR patients had higher pre-treatment immune-proteasome/ constitutive proteasome expression ratios [210] |
| Relapsed/refractory AML, FLT3-ITD/mutant, 1 month–21 years | FLT3 | Quizartinib | FLA + Etoposide | ITCC/COG | NCT03793478 | Phase I/II study |
| Relapsed/refractory AML, FLT3-ITD/mutant, 6 months–21 years | FLT3 | Gilteritinib | FLAG | Astellas | NCT04240002 | Phase I/II study |
| Children, adolescents, and young adults with newly-diagnosed FLT3-ITD or FLT3-mutant AML | FLT3 | Gilteritinib | Gilteritinib in combination with multi-agent chemotherapy | COG AAML1831 | NCT04293562 | Phase III trial |
| Relapsed/refractory AML, FLT3-ITD/mutant, 3–35 years | FLT3 | Pexidartinib |  | NCI | NCT02390752 |  |
| Relapsed/refractory AML, ≥12 years | FLT3 | MRX-2843 |  | Meryx | NCT04872478 |  |
| Relapsed/refractory AML, IDH2 mutation, 2–18 years | IDH2 | Enasidenib |  | COG | NCT04203316 |  |
| Relapsed/refractory AML, 1 month–21 years | NEDD8 | Pevonedistat | Azacytidine + FLA | COG/PEP-CTN | NCT03813147 |  |
| Relapsed/refractory AML, CNS^+/-^, 1–21 years | MDM2/MDI | ALRN-6924 | ± Cytarabine | DFCI | NCT03654716 |  |
| Relapsed/refractory AML, ≤30 years | MDM2 | Idasanutlin | FLA or Venetoclax | Hoffmann-La Roche | NCT04029688 |  |
| Relapsed/refractory AML | BCL-2 | Venetoclax | Venetoclax + high-dose Cytarabine and Idarubicin | VENAML phase I/II study | NCT03194932 | Determination of the recommended phase II dose (RP2D) [211] |
| Relapsed/refractory AML | BCL-2 | Venetoclax | Venetoclax + high-dose Cytarabine and Idarubicin | VENAML phase I/II study | NCT03194932 | Determination of the recommended phase II dose (RP2D) [211] |
|  |  |  |  |  |  |  |
| Relapsed/refractory AML, 2-20 years | BCL-2 | Venetoclax | Cytarabine ± Idarubicin | SJCRH | NCT03194932 |  |
| Relapsed/refractory CD33+ AML, 29 days–21 years | BCL-2 | Venetoclax | FLA + GO | LLS PedAL/EuPAL | NCT05183035 |  |
| Relapsed/refractory AML, ≤30 years | XPO1,  BCL-2 | Selinexor, Venetoclax | FLA/FLAG | SJCRH | NCT04898894 |  |
| Relapsed/refractory AML, CD33^+^ 1–35 years | CD33 | CD33 CAR-T cells | Fludarabine + Cyclophos- phamide LD | CIBMTR multisite | NCT03971799 |  |
| Relapsed/refractory AML, CD33^+^ ≤30 years | CD33 | CD33 CAR-T cells (DARIC) | Rapamycin (activates DARIC) | SCH | NCT05105152 |  |
| Relapsed/refractory AML, 2–21 years | CD33xCD3 | CD33xCD3 bispecific antibody |  | Y-mAbs therapeutics, COG/PEP-CTN | NCT05077423 |  |
| Relapsed/refractory AML | CD123xCD3 | Flotetuzumab |  | COG/PEP-CTN | NCT04158739 |  |
| Relapsed/refractory AML, CD123^+^,  1–29 years | CD123 | CD123 CAR-T cells | Fludarabine + Cyclophos  phamide LD, Rituximab for T cell termination | CHOP | NCT04678336 |  |
| Relapsed/refractory AML, ≥12 years | CD123 | SAR443579 (NK cell engager) |  | Sanofi | NCT05086315 |  |
| Relapsed/refractory AML, CLL-1^+^, ≤75 years | CLL-1 (CLEC12A, CD371) | CLL-1 CAR-T cells | Fludarabine + Cyclophos- phamide LD | BCM/TCH | NCT04219163 |  |
| Post-HSCT relapse, ≥1 year |  | CIML NK cells | FLAG or Fludara- bine + Cyclophos- phamide LD | WUSTL | NCT03068819 |  |
| Post-HSCT relapse, ≥1 year |  | CIML NK cells | Fludarabine + Cyclophos- phamide LD | WUSTL | NCT04024761 |  |
| Relapsed/refractory AML, KMT2A rear- rangement, NUP98 rear- rangement, or NPM1 mutation ≥30 days | Menin | Revumenib (SNDX-5613) |  | DFCI | NCT04065399 |  |
| Relapsed/refractory AML, KMT2A rear- rangement, NUP98 rear- rangement, or NPM1 mutation ≥30 days | Menin | Revumenib | FLA | Syndax | NCT05326516 |  |
| Relapsed/refractory AML, ≥12 years | Menin | Revumenib | Decitabine + Cedazuri- dine (ASTX727) + Vene- toclax | MDACC | NCT05360160 |  |
| Relapsed/refractory AML, 2–25 years | CREB | Niclosamide | Cytarabine | Stanford University | NCT05188170 |  |
| CBFA2T3::GLIS2 acute megakaryoblastic leukemia | Cell surface target protein *CBFA2T3::GLIS2* AML | FOLR1-directed ADC Luveltamab Tazevibulin | Fludarabine/Cytarabine, Decitabine, Methotrexate, or Dasatinib |  |  | Well-tolerated as monotherapy and in combination |

***AML****, acute myeloid leukemia****; ADC,*** *antibody-drug conjugates;* ***BCM/TCH****, Baylor College of Medicine/Texas Children’s Hospital;* ***CHOP****, Children’s Hospital of Philadelphia;* ***CIBMTR****, Center for International Blood and Marrow Transplant Research;* ***COG****, Children’s Oncology Group;* ***CREB****, cAMP response element-binding protein;* ***DARIC****, dimerizing agent-regulated immune-receptor complex;* ***DFCI****, Dana-Farber Cancer Institute;* ***FLA****, Fludarabine/Cytarabine;* ***FLAG****, Fludarabine/Cytarabine + G-CSF;* ***HSCT****, hematopoietic stem cell transplant;* ***ITCC****, Innovative Therapies for Childhood Cancer consortium;* ***LD****, lymphodepleting chemotherapy;* ***LLS PedAL/EuPAL****, Leukemia & Lymphoma Society Pediatric Acute Leukemia and European Pediatric Acute Leukemia consortium;* ***MDACC****, MD Anderson Cancer Center;* ***NCI****, National Cancer Institute;* ***GO****, Gemtuzumab Ozogamicin;* ***SCH****, Seattle Children’s Hospital;* ***SJCRH****, St Jude Children’s Research Hospital;* ***WUSTL****, Washington University in St Louis.*

207. Cooper TM, Absalon MJ, Alonzo TA, Gerbing RB, Leger KJ, Hirsch BA*, et al.* Phase I/II Study of CPX-351 Followed by Fludarabine, Cytarabine, and Granulocyte-Colony Stimulating Factor for Children With Relapsed Acute Myeloid Leukemia: A Report From the Children's Oncology Group. J Clin Oncol 2020;38 2170-2177.

208. van Eijkelenburg NKA, Rasche M, Ghazaly E, Dworzak MN, Klingebiel T, Rossig C*, et al.* Clofarabine, high-dose cytarabine and liposomal daunorubicin in pediatric relapsed/refractory acute myeloid leukemia: a phase IB study. Haematologica 2018;103 1484-1492.

209. Cooper TM, Alonzo TA, Gerbing RB, Perentesis JP, Whitlock JA, Taub JW*, et al.* AAML0523: a report from the Children's Oncology Group on the efficacy of clofarabine in combination with cytarabine in pediatric patients with recurrent acute myeloid leukemia. Cancer 2014;120 2482-2489.

210. Niewerth D, Kaspers GJ, Jansen G, van Meerloo J, Zweegman S, Jenkins G*, et al.* Proteasome subunit expression analysis and chemosensitivity in relapsed paediatric acute leukaemia patients receiving bortezomib-containing chemotherapy. J Hematol Oncol 2016;9 82.

211. Karol SE, Alexander TB, Budhraja A, Pounds SB, Canavera K, Wang L*, et al.* Venetoclax in combination with cytarabine with or without idarubicin in children with relapsed or refractory acute myeloid leukaemia: a phase 1, dose-escalation study. Lancet Oncol 2020;21 551-560.
